# Metabolite and Lipid Profiling of Biobank Plasma Samples Collected Prior to Onset of Rheumatoid Arthritis

**DOI:** 10.1371/journal.pone.0164196

**Published:** 2016-10-18

**Authors:** Izabella Surowiec, Lisbeth Ärlestig, Solbritt Rantapää-Dahlqvist, Johan Trygg

**Affiliations:** 1 Computational Life Science Cluster (CLiC), Department of Chemistry, Umeå University, Umeå, Sweden; 2 Department of Public Health and Clinical Medicine, Rheumatology, Umeå University Hospital, Umeå, Sweden; University of Birmingham, UNITED KINGDOM

## Abstract

**Objective:**

The early diagnosis of rheumatoid arthritis (RA) is desirable to install treatment to prevent disease progression and joint destruction. Autoantibodies and immunological markers pre-date the onset of symptoms by years albeit not all patients will present these factors, even at disease onset. Additional biomarkers would be of high value to improve early diagnosis and understanding of the process, leading to disease development.

**Methods:**

Plasma samples donated before the onset of RA were identified in the Biobank of Northern Sweden, a collection within national health survey programs. Thirty samples from pre-symptomatic individuals and nineteen from controls were subjected to liquid chromatography-mass spectrometry (LCMS) metabolite and lipid profiling. Lipid and metabolite profiles discriminating samples from pre-symptomatic individuals from controls were identified after univariate and multivariate OPLS-DA based analyses.

**Results:**

The OPLS-DA models including pre-symptomatic individuals and controls identified profiles differentiating between the groups that was characterized by lower levels of acyl-carnitines and fatty acids, with higher levels of lysophospatidylcholines (LPCs) and metabolites from tryptophan metabolism in pre-symptomatic individuals compared with controls. Lipid profiling showed that the majority of phospholipids and sphingomyelins were at higher levels in pre-symptomatic individuals in comparison with controls.

**Conclusions:**

Our LCMS based approach demonstrated that there are changes in small molecule and lipid profiles detectable in plasma samples collected from the pre-symptomatic individuals who subsequently developed RA, which point to an up-regulation of levels of lysophospatidylcholines, and of tryptophan metabolism, perturbation of fatty acid beta-oxidation and increased oxidative stress in pre-symptomatic individuals’ years before onset of symptoms.

## Introduction

Rheumatoid arthritis (RA) is a chronic inflammatory autoimmune disease which causes joint destruction, and progressively leads to a deterioration in the quality of life. Its etiopathogenesis has not been completely elucidated, but it is widely accepted that RA is a complex and multifactorial disease influenced by both genetic and environmental factors [1]. Early diagnosis is of importance to be able to initiate treatment to inhibit the progression of the disease [2]. Antibodies against citrullinated peptides (ACPA) related to disease progression appear very early in the course of the disease, even years before appearance of joint symptoms, and their levels increase close to the disease onset [3, 4]. During the pre-dating time before the onset of symptoms the frequency of these antibodies is around 30% increasing to approximately 60–70% after diagnosis of disease. Other biomarkers, such as cytokines and plasma lipids, also pre-date onset of symptoms as well as increased levels of C-reactive protein (CRP) and cholesterol [5–7]. However, there remains a need to explore the underlying pathogenic process leading to the development of the disease.

Metabolomics is focused on investigation of chemical processes involving metabolites. As such, metabolomics aims at quantification of all, or as many as possible metabolites in the given biological sample with subsequent statistical analysis of their levels in relation to the question of the study. Lipidomics has the same aims, with the only difference being that instead of low mass molecules one is focused on profiling lipid species extracted from the studied samples. Since small metabolites and lipids have different physico-chemical properties, their analysis requires different extraction and profiling protocols, although in both cases liquid chromatography-mass spectrometry (LCMS) can be used to separate and identify the compound species studied. In clinical studies profiling approaches are primarily aimed towards identification of biomarkers or biomarker patterns indicative of the physiological state and/or understanding of the pathology of the studied disease. Several studies have described the application of metabolomics in RA and were focused on finding metabolites as potential biomarkers for the disease [8–12], or for treatment effects [13, 14] and proposing metabolic pathways perturbed during RA.

To our knowledge, no studies have applied metabolomic and lipidomic investigations using plasma samples from pre-symptomatic individuals who subsequent develop RA stored as part of a biobank. The aim of our study was to identify biochemical patterns and pathways involved in the generation of disease before onset of symptoms, and to find a set of biomarkers that may be useful in making an early diagnosis of RA.

## Patients and Methods

### Samples

The register of patients attending the Department of Rheumatology, University Hospital of Umeå and who fulfilled the 1987 American Rheumatism Association classification criteria for RA [15] were co-analysed with the registers of population based cohorts of the Medical Biobank of Northern Sweden. These registers include participants in the Västerbotten intervention project, the WHO study for Monitoring of Trends and Determinants in cardiovascular disease (Monica) project, and the Mammography Screening project including women invited for mammography. The criteria concerning the recruitment, blood sampling and storage condition (-80°C) have been described previously [3]. The study was approved by the Regional Ethics Committee of the University Hospital in Umeå. A cohort of 479 individuals before any symptoms of joint disease who have subsequently developed RA and having donated a total of 512 samples was identified. For this study only females (n = 326) with BMI <30 kg/m^2^, being drug free, non-smoking and fasting (thus excluding samples from the Mammography project)with blood samples taken up to seven years before the onset of any symptoms of the disease were included. Originally we planned for having two pre-symptomatic individuals and one control in each pair (40 +20), but exclusion criteria resulted in the selection of a minor cohort of 30 pre-symptomatic individuals and 19 controls. The individuals are referred to as “pre-symptomatic individuals”. Control subjects were randomly selected from the same cohorts within the Medical Biobank as the pre-symptomatic individuals, and matched for sex, age and sampling time on group level. The median (interquartile range (IQR)) time pre-dating the onset of symptoms of RA was 4.7 (3.0) years. Selected clinical and laboratory characteristics of patients who had provided the blood samples included in this study are presented in Table 1. Positivity for ACPA was tested using anti-cyclic citrullinated peptides (anti-CCP2) test, according to manufactures instructions (Euro Diagnostica, Malmö, Sweden) with cut-off set at 25 AU/mL. ApoA1 (g/L) and ApoB (g/L) were analysed using immunoturbimetric method on a Cobas 8000 instrument (Roche Diagnostics Scandinavia AB).

**Table 1:** Descriptive data for pre-symptomatic individuals with plasma samples pre-dating the onset of RA by a median of 4.7 years and for control subjects.

Variables	Pre-symptomatic individuals (n = 30)	Controls (N = 19)
**Age (mean)**± **SD, years**	54.5±6.2	51.5±9.5
**HLA-SE**^**1**^**, *n* (%)**	20 (71.4)	-
**ACPA+**^**2**^**, *n* (%)**	11 (35.5)	-
**Smoking, ex, *n* (%)**	14 (48.3)	6 (33.3)
**BMI kg/m**^**2**^***(mean±SD)***	24.4±2.7	24.3±4.3
**ApoA1, g/L *(mean±SEM)***	1.57±0.03	1.47±0.06
**ApoB, g/L *(mean±SEM)***	1.08±0.05	1.03±0.05
**ApoB/ApoA1**	0.70	0.73

1 HLA-SE = HLA shared epitope = 0101/0401/0404/0405/0408

2 Analysed using anti-CCP2 antibody test

### Metabolomics analysis

Plasma was thawed on ice and 630 μL of extraction mixture (H_2_O:methanol (1:9, v/v)) were added to 70 μL of plasma; extraction of metabolites was then carried out using a MM301 vibration Mill (Retsch GmbH & Co. KG, Haan, Germany) at a frequency of 30 Hz for 2 min. Samples were stored on ice for 2 h to allow protein precipitation, after which they were centrifuged at 18 000 rcf for 10 min at 4°C. An aliquot (200 μL) of the resulting supernatant was transferred to a liquid chromatography vial and evaporated to dryness at room temperature in a miVac QUATTRO concentrator (Genevac LTD, Ipswich, UK). Samples were then dissolved in 20 μL of methanol:water (1:1, v/v) mixture and 2 μL analysed with LCMS system as described in S1 File.

### Lipidomics analysis

Plasma was thawed on ice and 110 μL of extraction mixture (chloroform:methanol (2:1, V/V) was added to 20 μL of plasma and extraction carried out using a MM301 vibration Mill (Retsch GmbH & Co. KG, Haan, Germany) at a frequency of 30 Hz for 2 min. Then samples were stored at ambient temperature for 60 min before being centrifuged at 14 000 rpm for 3 min at 4°C. A 50 μL aliquot of the resulting lower phase was transferred to a LC vial, 70 μL of a chloroform:methanol (2:1, V/V) mixture were added and the samples shaken briefly before being analysed by LCMS as described in S1 File.

### Compound identification

Targeted Feature Extraction of the acquired LC-MS data was performed using the Profinder™ software package, version B.06.00 (Agilent Technologies Inc., Santa Clara, CA, USA) and an in-house retention time and mass spectra library consisting of 713 metabolites or 487 lipid species. Feature detection was based on following parameters: allowed ion species in positive ionization mode: (+H, +Na, +K, +NH_4_); in negative ionization mode: (-H, +HCOO) peak spacing tolerance: 0.0025–7 ppm; isotope model: common organic molecules; charge state: 1; mass tolerance: 10 ppm; retention time tolerance: 0.1 min. After extraction of peaks, each compound was manually checked for mass and retention time agreement with appropriate standards from the library; peaks with bad characteristics (overloaded, noisy, non- Gaussian, *etc*.) were excluded from the analysis. Identification of the compounds was confirmed by comparison of their MS/MS spectra with MS/MS spectra of relevant standards. For LCMS analysis of metabolites, combined data set was used, with compounds detected in both negative and positive ion modes. If a metabolite was detected in both modes, the one with higher signal intensity was retained for statistical analysis. In the lipidomics study only the positive mode was used.

### Data processing and multivariate and univariate data analysis

Compound data were imported into the SIMCA software (version 14.0) from MKS Umetrics AB (Umeå, Sweden) for multivariate analysis. All data were mean centred and scaled to unit variance. Principal component analysis (PCA) was used to check if there were any outliers among analysed samples. Seven-fold cross-validation was used for calculating in all models. Orthogonal partial least squares discriminant analysis (OPLS-DA) was used to compare metabolite profiles of different classes of samples; 1+1 component models were used to avoid the risk of over-fitting [16]. The significance of a metabolite for classification in the OPLS-DA models was specified by calculating the 95% confidence interval for the loadings using jack-knife [17].

Univariate analysis was done with GraphPad Prism 6 (San Diego, CA, USA) to check the normality distribution for each metabolite and to detect significant differences between pre-symptomatic individuals and controls. Significant differences were assessed using unpaired t-test with Welch’s correction and Wilcoxon t-test for normal and not normal distribution at p < 0.05, respectively. Receiver operating characteristic (ROC) curves [18] for significant metabolites were also calculated to evaluate predictive capacity of each metabolite to differentiate between pre-symptomatic RA individuals and controls. The Pearson correlations between metabolite and lipid levels and health survey data from VIP/MONICA-WHO cohort was done using were calculated using in-house script written using the Anaconda Python distribution v. 3.5 (https://continuum.io). The Pearson correlation coefficients and the corresponding p-value against the null-hypothesis of no correlation were calculated using functions from the SciPy library (http://www.scipy.org/). The results were plotted using the Matplotlib library (http://matplotlib.org/).

All data used in this study are available in the S2 File.

## Results

### Correlation between metabolite and lipids levels and health survey data

**Fig 1:**
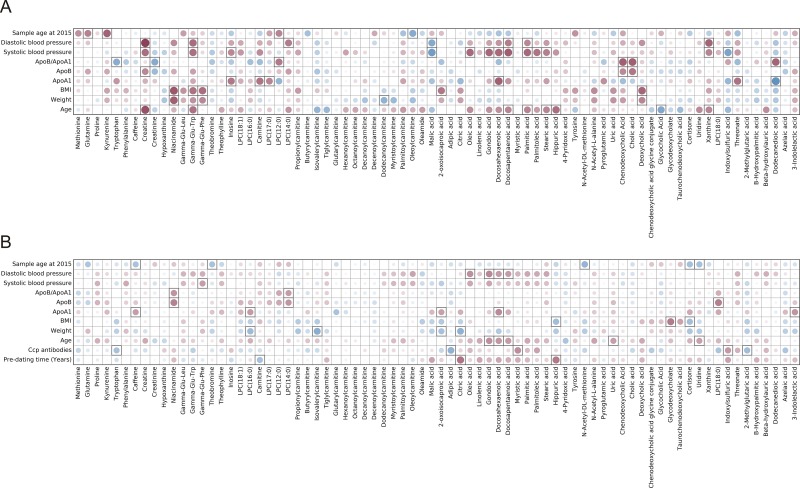
Plot showing correlation between health survey data and metabolite levels for A) controls and B) pre-symptomatic individuals, the color and size of the circles correspond to the strength of the correlation, with increasing circle size and color intensity indicating increasing correlation; shades of blue are used for negative correlations and shades of red for positive correlations, squares indicate correlations that were statistically significant (p-value < 0.05).

**Fig 2:**
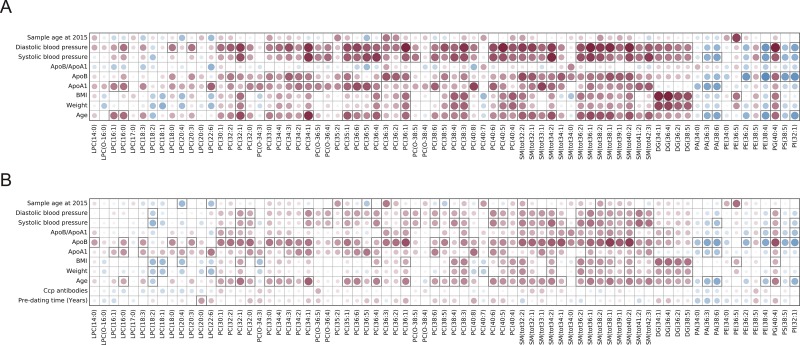
Plot showing correlation between health survey data and selected lipid levels for A) controls and B) pre-symptomatic individuals; the color and size of the circles correspond to the strength of the correlation, with increasing circle size and color intensity indicating increasing correlation; shades of blue are used for negative correlations and shades of red for positive correlations, squares indicate correlations that were statistically significant (p-value < 0.05), and (ii) the upper panel shows the corresponding Pearson’s correlation coefficient r.

**Fig 3:**
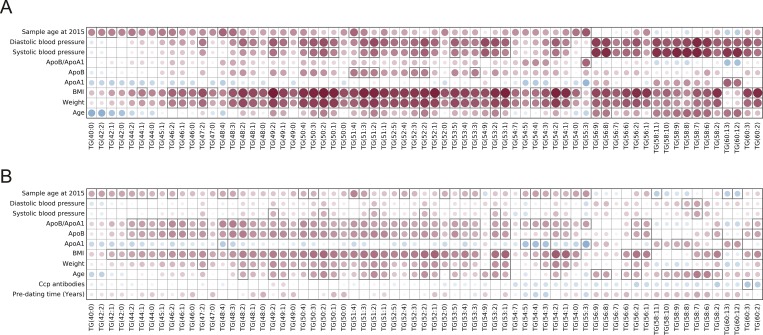
Plot showing correlation between health survey data and levels of triglycerides detected with LC-MS approach for A) controls and B) pre-symptomatic individuals; the color and size of the circles correspond to the strength of the correlation, with increasing circle size and color intensity indicating increasing correlation; shades of blue are used for negative correlations and shades of red for positive correlations, squares indicate correlations that were statistically significant (p-value < 0.05).

We have correlated lipid and metabolite levels detected with LCMS method in both pre-symptomatic individuals and controls with the available data collected from the health surveys performed when the samples were collected. Obtained results are presented as Figs 1–3. When metabolic data were considered, there were not so many significant correlations with the data from the health surveys. Of interest was that presence of ACPA was significantly positively correlated with myristic acid, indoxylsulfuric acid, whereas negatively with tryptophan levels, (p = 0.019, p = 0.027 and p = 0.014, respectively). There were many significant correlations between the identified lipid species and available personal parameters (*e*.*g*., age, weight, BMI, blood pressure, lipoprotein levels, etc.). Lipid species were as expected positively correlated with the person’s age, weight, BMI, levels of blood sugar and blood pressure for both controls and pre-symptomatic individuals (Figs 2 and 3). Differences were, however, observed for correlations of lipid levels with levels of ApoB, ApoA1 and their ratios. Triacylglycerides detected with LCMS method showed negative correlation with ApoA1 levels and positive with both ApoB and ApoB:ApoA1 ratio, although significant for majority of triglycerides only in pre-symptomatic individuals (Fig 3). Other classes of lipids were for the majority of compounds positively correlated with both ApoB, ApoA1 and ApoB:ApoA1 ratio, but also in this case the last correlation was stronger for pre-symptomatic individuals (Fig 2).

### Metabolite profiling

**Fig 4:**
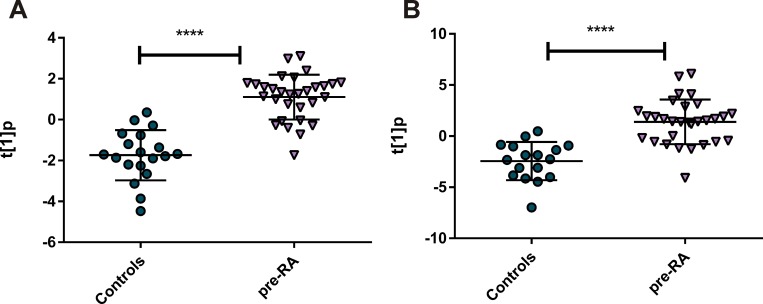
OPLS-DA score plots of the first predictive component (t[1]p) showing the separation between pre-symptomatic individuals and controls; A) LCMS metabolomics profiling (p < 0.0001), B) LCMS lipidomics profiling (p < 0.0001).

**Fig 5:**
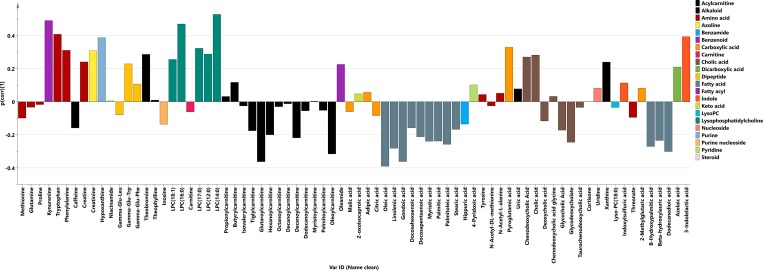
Metabolite predictive loading values (p(corr)) in the OPLS-DA model between pre-symptomatic individuals and controls; metabolites are coloured according to their chemical classes; p(corr) values indicate if the metabolite is in higher or lower levels in pre-symptomatic individuals in comparison with controls.

Principal component analysis (PCA) was used to achieve an overview of all metabolite data from the 49 samples and the 76 identified metabolites (Table A in S1 File). The resulting six-component model, R^2^X(cum) = 0.525, showed no outliers in the data (data not shown). Next OPLS-DA model between pre-symptomatic individuals (n = 30) and controls (n = 19) was calculated with one predictive and one orthogonal components, with 4.6% of variation in the data explained by predictive component (Fig 4A). Metabolite profile (p(corr)) connected to the pre-symptomatic individuals is presented at Fig 5. Significant metabolites according to the jack-knife confidence intervals were: oleic acid (lower levels in pre-symptomatic individuals compared with controls), as well as kynurenine and LPC(16:0) (higher levels in pre-symptomatic individuals in comparison to controls). Whole profile was also characterized by lower levels of majority of acyl-carnitines and fatty acids in pre-symptomatic individuals and LPCs and aromatic amino acids up-regulated in pre-symptomatic individuals compared with controls.

According to univariate statistics, the following compounds were significantly different between samples from the pre-symptomatic individuals and controls: oleic acid (p = 0.040, AUROC = 0.67), β-hydroxypalmitic acid (p = 0.049, AUROC = 0.67), kynurenine (p = 0.015, AUROC = 0.66), hypoxanthine (p = 0.044, AUROC = 0.69), LPC(16:0) (p = 0.049, AUROC = 0.69), LPC(14:0) (p = 0.012, AUROC = 0.70) and 3-indolelactic acid (p = 0.045, AUROC = 0.65). Distribution of the levels of significant compounds and their ROC curves for differentiation between pre-symptomatic individuals and controls are presented in Figure A in S1 File.

### Lipid profiling

**Fig 6:**
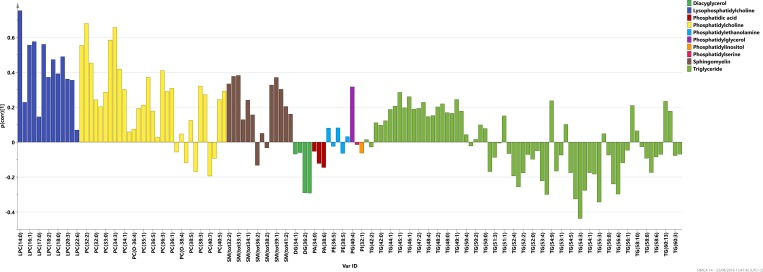
Lipid profiling predictive loading values (p(corr)) in the OPLS-DA model between pre-symptomatic individuals and controls; lipid species are coloured according to their chemical classes; p(corr) values indicate if the metabolite is in higher or lower levels in pre-symptomatic individuals in comparison with controls.

Principal component analysis (PCA) was used to get an overview of all metabolite data from the 49 samples and 140 identified lipid species (Table B in S1 File). The resulting five-component model, R^2^X(cum) = 0.786, showed two outliers in the data, both belonging to the control group and characterized by higher levels of all lipids compared with the remaining samples (figure not shown). These samples were excluded from further analysis and a new PCA 8-component model made on the 47 other samples (R^2^X(cum) = 0.82) revealed no further outliers (data not shown). Subsequently, an OPLS-DA model between pre-symptomatic individuals (n = 30) and controls (n = 17) was calculated with one predictive and one orthogonal component with 6.6% of variation in the data being explained by the predictive component (Fig 4B). The lipid profile (p(corr)) connected to the pre-symptomatic individuals is presented in Fig 6. Significant metabolites according to the jack-knife confidence intervals were: LPCs (14:0), (16:0), (16:1), (18:1), (18:3), (20:4), (20:3); phospchocholines: (30:1), (32:1), (32:2), (34:2), (34:4), (O-34:3) and sphingomyelins: (33:1), (32:1), (38:1), (39:1). The whole profile was characterized by more lipids being detected at higher levels in the pre-symptomatic individuals in comparison to controls, especially those belonging to classes of LPCs, phsophatidylcholines and sphingomyelines.

According to univariate statistics, the following compounds were significantly different between samples from pre-symptomatic individuals and controls: LPC(16:0) (p = 0.027, AUROC = 0.7), LPC(14:0) (p = 0.0008, AUROC = 0.76), LPC(18:3) (p = 0.025, AUROC = 0.64), PC(30:1) (p = 0.038, AUROC = 0.38), PC(32:2) (p = 0.005, AUROC = 0.73), PC(34:3) (p = 0.010, AUROC = 0.73) and PC(34:4) (p = 0.047, AUROC = 0.67), all in higher levels in pre-symptomatic individuals compared with controls. Distribution of levels of the significant compounds and their ROC curves for differentiation between pre-symptomatic individuals and controls are presented in Figure A in S1 File.

## Discussion

Both metabolic and lipidomic models comparing pre-symptomatic individuals and controls were valid according to an applied cross-validation procedure, with limited number of compounds being significantly different between two groups of samples according both to jack-knife confidence intervals and to the univariate statistics performed by t-test and AUROC analysis (AUROC values of significant compounds were around 0.7, indicating their poor to fair diagnostic value). Presented results indicate that differences between pre-symptomatic individuals and controls, as detected using LCMS profiling, were small. This could be expected, since the samples from the pre-symptomatic individuals analysed were taken up to seven years before the onset of any symptoms of the disease and, hence, the pathogenic processes could be at a very early stage. However, the results obtained show that there is a difference in metabolic profiles between pre-symptomatic individuals and controls which can be evaluated using the chosen methodology. The potential biological context of the observed difference will be discussed.

Two LPC’s ((14:0) and LPC (16:0)) were detected with both platforms and were found to be significantly higher in the pre-symptomatic individuals compared with controls (Figures A and B in S1 File). Other LPC’s were also significantly increased according to the lipidomic OPLS-DA model between pre-symptomatic individuals and controls. LPCs are plasma lipids created from phospatidylcholines either by the action of phospholipase A_2_ or by oxidation. They have been recognized as important cell signalling molecules, involved in wide range of physiological and pathophysiological processes including cell proliferation, wound healing, muscle contraction, reproduction, neuronal diseases, sepsis and cancer [19–21]. They are also important inflammatory mediators with recognized effects in multiple immune cell types and, hence, connected to both innate and adaptive immune responses [22, 23]. Lysophosphatidylcholines can activate a number of secondary messengers by the vast amount of signalling pathways described in several reviews [24–28]. Lysophosphatidylcholines are also major components in oxLDLs and consequently are believed to play a vital role in atherosclerosis [29–31]. The ratio of phospholipids to corresponding LPC has been suggested to be a potential parameter for estimating inflammatory activity and monitoring clinical therapies [32], also in RA-patients [8]. Our findings would then point to the involvement of LPCs in the pathogenesis of RA via activation and modulation of the immune system. Furthermore, increased levels of LPCs could connect them to the increased risk for cardiovascular diseases observed in patients with RA [33, 34].

Kynurenine and 3-indolelactic acid were found to be significantly increased in the pre-symptomatic individuals compared with control subjects. Both are degradation products of tryptophan from two major pathways by which this molecule is metabolized in humans (kynurenine pathway or via a series of indoles, respectively). Tryptophan was also one of the metabolites with highest p(corr) values in an OPLS-DA model between pre-symptomatic individuals and controls. There were significantly lower levels of tryptophan in ACPA positive pre-symptomatic individuals compared with ACPA negative ones, which could suggest a link of this molecule with up-starting inflammatory processes. Our results indicate an increased metabolism of tryptophan that can most probably be related to the biological activity of its metabolites, especially in connection with immune activation. Degradation of tryptophan in the kynurenine pathway is initiated by the cleavage of an indole ring via indoleamine 2,3-dioxygenase (IDO), an extrahepatic enzyme induced by cytokines, mainly interferon-γ (IFN-γ). Increased kynurenine/tryptophan ratio is a marker of IDO activity and a cell-mediated immune response [35]. Metabolites of the kynurenine pathway take part in homeostasis of the immune response by playing an active role in suppressing T-cell proliferation with apoptosis being the proposed mechanism [36]. In particular, they have been directly connected to the regulation of levels of pro-inflammatory Th17 cells, which have been found to be elevated in rheumatoid arthritis [37,38]. This would explain the adverse health effects observed after blockage of the kynurenine pathway in mice models of RA [39, 40], and the correlation of tryptophan degradation with the severity of the disease in humans [41]. Consequently there is an increased interest in the possibility of exploring this pathway as being therapeutic in cases of autoimmune diseases [37]. Several studies have reported reduced levels of tryptophan and increased levels of kynurenine (an increased kynurenine:tryptophan ratio) in patients with RA in comparison to control subjects [41–43], confirming activation of kynurenine pathway in RA as an attempt (albeit not sufficiently effective) to down-regulate the immune system. In our study there were, however, no significant differences in kynurenine:tryptophan ratios between pre-patients and controls (data not shown). Furthermore, in our study tryptophan levels were higher in pre-symptomatic individuals contradicting the findings reported for active RA patients [41–43], but in agreement with results from the analysis of synovial fluid from active RA patients [9]. Lower kynurenine:tryptophan ratio was also found in pre-arthritic mice compared with ones that have not developed arthritis [37], what would suggest that high ratios of these compounds are typical for active disease. This could explain why we did not observed increased kynurenine/tryptophan ratios in pre-symptomatic RA patients when compared to controls in our study. In this study in which we analysed plasma samples from individuals several years before onset of the disease the results would suggest a lack of increased activity of IDO, or activation of the compensation for tryptophan degradation that manages to restrain depletion of its levels in plasma. More work should be done to investigate mechanisms behind the observed changes, but still the results of our study point to the initiation of inflammatory processes in patients with RA long before any apparent clinical symptoms.

Increased levels of hypoxanthine in pre-symptomatic individuals compared with control subjects observed in our data could be connected to changes in xanthine oxidase (XO) activity—an enzyme known for its production of reactive oxygen species that has been related to an increased inflammatory response and oxidative stress in many diseases [44] including RA [45, 46]. Xanthine oxidase catalyses the oxidation of hypoxanthine to xanthine and can further catalyse oxidation of xanthine to uric acid. In our study all of the mentioned metabolites were present at higher levels in pre-symptomatic individuals compared with controls. Activation of XO would result in increased xanthine:hypoxanthine and uric acid:xanthine ratios. However, in our study there were no significant differences in either of these ratios between the pre-symptomatic individuals and controls. Upregulated levels of hypoxanthine and xanthine could be connected to increased catabolism of nucleotides in, for example, hypoxia conditions. In fact, plasma hypoxanthine has been proposed to be a good indicator of tissue hypoxia [47], and has been reported to be increased in plasma from RA patients in comparison with controls as a result of leakage of this molecules from the inflamed ischaemic joints [12, 48].

Fatty acids and acylcarnitines, both of which are connected to β-oxidation taking place in mitochondria, were down-regulated in the pre-symptomatic individuals compared with controls. Such a profile could be explained by the increased turn-over through the β-oxidation pathway in relation to an enhanced energy demand, caused, for example, by an inflammatory response, and/or in connection to the production of reactive oxygen species [49]. Fatty acid oxidation in macrophages has also been described to be related to an anti-inflammatory response [50]. Lowered levels of fatty acids could also point on decreased lipolysis, observed for example in SLE patients [51]. Lower fatty acid levels were also detected in analysis of synovial fluid from active patients as compared with other types of inflammatory arthritis [9]. However, because fatty acids play a central role in human metabolism and can be connected to different processes (*e*.*g*., lipolysis, lipogenesis, beta-oxidation, *etc*.), more work is needed to understand the biological significance of changes in their levels in pre-symptomatic RA individuals.

Significant positive correlations between ApoB levels and ApoB:ApoA1 ratio with majority of detected triglycerides in pre-symptomatic individuals but not with controls could be connected to differences in lipoprotein metabolism between both studied groups, with more lipids being associated with ApoB levels. Our results would then be in line with the ones published by van Halm and others [6], who found more atherogenic lipid profile in pre-symptomatic RA patients as compared to controls already 10 years before disease onset. This profile was characterized by higher total cholesterol, triglyceride and ApoB levels and lower HDLc levels in pre-symptomatic individuals compared to controls. Still, more work is needed to understand the role of specific lipid species and fluctuations of their levels in the pre-symptomatic RA patients.

Only one study presenting investigation of early processes connected to the development of RA was reported [10]. In that study metabolomics analysis of plasma samples from patients with early RA was undertaken showing that metabolic profiles were able to stratify patients within this group according to the level of current inflammation as measured with CRP levels. Lactate and lipids were important discriminators of inflammatory burden in early arthritis, what is in agreement with our findings showing that inflammation related changes in metabolite and especially lipid metabolism can be detected already at the early stages of the disease.

## Conclusions

In conclusion, presented manuscript summarizes results from metabolomics and lipidomics analysis of biobank samples from individuals who subsequently developed RA. Samples from pre-symptomatic individuals are very rare, hence presented study is a worldwide unique example of investigation of metabolic processes taking place in humans before disease onset. We have been able to show that it is possible to detect differences in the metabolic and lipid profiles of plasma from biobank samples analysed years before the onset of symptoms for RA in comparison with control subjects. Our results also indicate that metabolic profiling is a potential tool to study mechanisms related to the pathogenesis of RA. The main metabolic and lipid differences observed point to a perturbation in lipid and tryptophan metabolism and can be connected to the changes in the level of oxidative stress. These findings add information to an emerging theoretical framework concerning the biological origin of RA and suggest potential additional biomarkers that could be used with existing ones for an early diagnosis of RA.

## Supporting Information

S1 File Supplementary Methods. Figure A. Distribution of levels of the significant compounds and their ROC curves for differentiation between pre-RA patients and controls as detected with LCMS metabolomics method. Figure B. Distribution of levels of the significant compounds and their ROC curves for differentiation between pre-RA patients and controls as detected with LCMS lipidomics method. Table A. Metabolites detected in plasma biobank samples investigated in the study. Table B. Lipid species detected in plasma biobank samples investigated in the study.
(DOCX)

S2 File Metabolomics and lipidomics data. (XLSX)
