# Supplementary material for: Metabolite and Lipid Profiling of Biobank Plasma Samples Collected Prior to Onset of Rheumatoid Arthritis
Source: PLoS One. 2016 Oct 18;11(10):e0164196. doi: 10.1371/journal.pone.0164196 (PMC5068821; doi:10.1371/journal.pone.0164196)
Supplement: S1 File — Figure A. Distribution of levels of the significant compounds and their ROC curves for differentiation between pre-RA patients and controls as detected with LCMS metabolomics method. Figure B. Distribution of levels of the significant compounds and their ROC curves for differentiation between pre-RA patients and controls as detected with LCMS lipidomics method. Table A. Metabolites detected in plasma biobank samples investigated in the study. Table B. Lipid species detected in plasma biobank samples investigated in the study. (DOCX) [file pone.0164196.s001.docx]

**Metabolite and lipid profiling of biobank plasma samples collected prior to onset of rheumatoid arthritis**

*Izabella Surowiec, Lisbeth Ärlestig, Solbritt Rantapää-Dahlqvist, Johan Trygg*

**Supporting Information**

**Supplementary Methods**

*LC-MS metabolomics analysis*

Aliquots of samples (2 µL) were injected into an Agilent UPLC system (Infinity 1290) equipped with a UPLC column (Acquity HSS T3, 2.1 x 50 mm, 1.8 µm C18 in combination with a 2.1 mm x 5 mm, 1.8 µm VanGuard precolumn (Waters Corporation, Milford, MA, USA). The UPLC system was coupled to an Agilent 6550 iFunnel Jet stream electrospray ion source Accurate-Mass QTOFMSMS (Agilent Technologies, Santa Clara, CA, USA). The mobile phases used were MilliQ water containing 0.1 % formic acid (A) and a mixtures of 75:25 acetonitrile: 2-propanol with 0.1 % formic acid (B). The following gradient was used: 0.1 - 10 % B for 2 min at a flow rate of 0.5 mL/min, then B was increased to 99 % during 5 min and held at 99 % for 2 min, B was then decreased to 0.1 % during 0.3 min and the flow-rate was increased to 0.8 mL/min for 0.5 min; these conditions were maintained for 0.9 min after which the flow-rate was reduced to 0.5 mL/min for 0.1 min before injection of the subsequent sample. Column oven temperature was held at 40°C whilst samples were kept at 5°C in the auto sampler at. Analysis was initially performed in the positive mode for all samples and subsequently in the negative mode using a second injection of each sample. Mass spectrometry parameters, with exception of the capillary voltage, were kept identical between the modes

A reference interface was connected to ensure accurate mass measurements; the reference ions purine (4 µM) and HP-0921 (Hexakis(1H, 1H, 3H-tetrafluoropropoxy)phosphazine) (1 µM) both purchased from Agilent Technologies (Santa Clara, CA, USA) were infused directly into the mass spectrometer at a flow rate of 0.05 mL/min for internal calibration, and the monitored ions were purine m/z 121.05 and m/z 119.03632; HP-0921 m/z 922.0098 and m/z 966.000725 for positive and negative mode, respectively. The gas temperature was set to 150°C, the drying gas flow to 16 L/min and the nebulizer pressure 35 psi. The sheath gas temp was set to 350°C and the sheath gas flow to 11 L/min. The capillary voltage was set to 4000 V in positive ion mode, and to 4500 V in negative ion mode. The nozzle voltage was 300 V. The “fragmentor” voltage was 380 V, the skimmer 45 V and the OCT 1 RF Vpp 750 V. The collision energy was set to 0 V. The m/z range was 70 - 1700, and data were collected in centroid mode with an acquisition rate of 4 scans/s.

*LC-MS lipidomics analysis*

An aliquot (2 µL) of the sample was injected into an Agilent UPLC system (Infinity 1290) equipped with a UPLC column (Acquity CSH, 2.1 x 50 mm, 1.7 µm C18 in combination with a 2.1 mm x 5 mm, 1.7 µm VanGuard CSH precolumn; Waters Corporation, Milford, MA, USA). The UPLC system was coupled to an Agilent 6540 iFunnel Jet stream electrospray ion source QTOFMSMS (Agilent Technologies, Santa Clara, CA, USA). Mobile phases used were 60:40 ACN:water + 10 mM ammonium formate + 0.1% formic acid (A) and 89.1:10.5:0.4 IPA:ACN:water + 10 mM ammonium formate + 0.1% formic acid (B). The following gradient was used: 0.0 - 15 % B at a flow rate of 0.5 mL/min, then B was increased to 30 % during 1.2 min, then to 55% during 0.3 min and held at 55 % for 3.5 min, then to 72% during 2 min, to 85% during 2.5 min and to 100% during 0.5 min where was held at 0.5 ml/min flow rate for additional 2 min after which the flow rate was increased to 5ml/min and kept for 0.5 min to wash the injection valve. After 12 min data was no longer acquired by the mass spectrometer. The column was equilibrated with 15% B for 1.5 min at a flow rate of 0.5 ml/min before the next sample was injected. The column oven temperature was held at 65°C and samples were kept in auto sampler at 10°C. Analysis was performed in the positive ion mode. A reference interface was connected to facilitate accurate mass measurements; both of the reference ions (purine (4 µM) and HP-0921 (Hexakis(1H, 1H, 3H-tetrafluoropropoxy)phosphazine) (1 µM) were purchased from Agilent Technologies (Santa Clara, CA, USA)) were infused directly into the MS at a flow rate of 0.08 mL/min for internal calibration, and the monitored ions were purine m/z 121.05 and m/z 119.03632; HP-0921 m/z 922.0098 and m/z 966.000725 for positive and negative mode, respectively. The gas temperature was set to 300°C, the drying gas flow to 8 L/min and the nebulizer pressure 40 psi. The sheath gas temp was set to 350°C and the sheath gas flow to 11 L/min. The capillary voltage was set to 4000 V in positive ion mode. The nozzle voltage was 0 V. The fragmentor voltage was 100 V, the skimmer 45 V and the OCT 1 RF Vpp 750 V. The collision energy was set to 0 V. The m/z range was 70 - 1700, and data were collected in centroid mode with an acquisition rate of 4 scans/s.

**Figures**

|  |  |  |  |
| --- | --- | --- | --- |
|  |  |  |  |
|  |  |  |  |
|  |  |  |  |

Figure A. Distribution of levels of the significant compounds and their ROC curves for differentiation between pre-RA patients and controls as detected with LCMS metabolomics method.

|  |  |  |  |
| --- | --- | --- | --- |
|  |  |  |  |
|  |  |  |  |
|  |  |  |  |

Figure B. Distribution of levels of the significant compounds and their ROC curves for differentiation between pre-RA patients and controls as detected with LCMS lipidomics method.

**Tables**

Table A. Metabolites detected in plasma biobank samples investigated in the study.

| **Compound name** | **Metabolite class** | **Molecular formula** | **Molecular mass** | **CAS number** | **HMDB number** |
| --- | --- | --- | --- | --- | --- |
| Methionine | Amino acid | C5 H11 N O2 S | 149.051 | 63-68-3 | HMDB00696 |
| Glutamine | Amino acid | C5 H10 N2 O3 | 146.069 | 56-85-9 | HMDB00641 |
| Proline | Amino acid | C5 H9 N O2 | 115.064 | 147-85-3 | HMDB00162 |
| Kynurenine | Benzenoid | C10 H12 N2 O3 | 208.085 | 2922-83-0 | HMDB00684 |
| Tryptophan | Amino acid | C11 H12 N2 O2 | 204.09 | 73-22-3 | HMDB00929 |
| Phenylalanine | Amino acid | C9 H11 N O2 | 165.079 | 150-30-1 | HMDB00159 |
| Caffeine | Alkaloid | C8 H10 N4 O2 | 194.08 | 58-08-2 | HMDB01847 |
| Creatine | Amino acid | C4 H9 N3 O2 | 131.069 | 57-00-1 | HMDB00064 |
| Creatinine | Azoline | C4 H7 N3 O | 113.059 | 60-27-5 | HMDB00562 |
| Hypoxanthine | Purine | C5 H4 N4 O | 136.039 | 68-94-0 | HMDB00157 |
| Niacinamide | Pyridine | C6 H6 N2 O | 122.048 | 98-92-0 | HMDB01406 |
| Gamma-Glu-Leu | Dipeptide | C11 H20 N2 O5 | 260.137 | 2566-39-4 | HMDB11171 |
| Gamma-Glu-Trp | Dipeptide | C16 H19 N3 O5 | 333.134 | 66471-20-3 | NA |
| Gamma-Glu-Phe | Dipeptide | C14 H18 N2 O5 | 294.122 | 7432-24-8 | HMDB00594 |
| Theobromine | Alkaloid | C7 H8 N4 O2 | 180.065 | 83-67-0 | HMDB02825 |
| Theophylline | Alkaloid | C7 H8 N4 O2 | 180.065 | 58-55-9 | HMDB01889 |
| Inosine | Purine nucleoside | C10 H12 N4 O5 | 268.081 | 58-63-9 | HMDB00195 |
| LPC(18:1) | LysoPC | C26 H52 N O7 P | 521.348 | 19420-56-5 | HMDB10408 |
| LPC(16:0) | LysoPC | C24 H50 N O7 P | 495.333 | 17364-16-8 | HMDB10382 |
| Carnitine | Carnitine | C7 H15 N O3 | 161.105 | 541-15-1 | METPA0048 |
| LPC(17:0) | LysoPC | C25 H52 N O7 P | 509.349 | 50930-23-9 | HMDB12108 |
| LPC(12:0) | LysoPC | C20 H42 N O7 P | 439.27 | 20559-18-6 | NA |
| LPC(14:0) | LysoPC | C22 H46 N O7 P | 467.301 | 20559-16-4 | HMDB10379 |
| Propionylcarnitine | Acylcarnitine | C10 H19 N O4 | 217.131 | 17298-37-2 | HMDB00824 |
| Butyrylcarnitine | Acylcarnitine | C11 H21 N O4 | 231.147 | 25576-40-3 | HMDB02013 |
| Isovalerylcarnitine | Acylcarnitine | C12 H23 N O4 | 245.163 | 31023-24-2 | HMDB00688 |
| Tiglylcarnitine | Acylcarnitine | C12 H21 N O4 | 243.147 | 64191-86-2 | HMDB02366 |
| Glutaroylcarnitine | Acylcarnitine | C12 H21 N O6 | 275.137 | 102636-82-8 | NA |
| Hexanoylcarnitine | Acylcarnitine | C13 H25 N O4 | 259.178 | 6418-78-6 | HMDB00705 |
| Octanoylcarnitine | Acylcarnitine | C15 H29 N O4 | 287.21 | 25243-95-2 | HMDB00791 |
| Decanoylcarnitine | Acylcarnitine | C17 H33 N O4 | 315.241 | 1492-27-9 | HMDB00651 |
| Decenoylcarnitine | Acylcarnitine | C17 H31 N O4 | 313.225 | Not available | NA |
| Dodecanoylcarnitine | Acylcarnitine | C19 H37 N O4 | 343.273 | 25518-54-1 | HMDB02250 |
| Myristoylcarnitine | Acylcarnitine | C21 H41 N O4 | 371.304 | 25597-07-3 | HMDB05066 |
| Palmitoylcarnitine | Acylcarnitine | C23 H45 N O4 | 399.336 | 2364-67-2 | HMDB40367 |
| Oleoylcarnitine | Acylcarnitine | C25 H47 N O4 | 425.351 | 38677-66-6 | HMDB05065 |
| Oleamide | Fatty acyl | C18 H35 N O | 281.272 | 301-02-0 | HMDB02117 |
| Malic acid | Carboxylic acid | C4 H6 O5 | 134.022 | 6915-15-7 | HMDB00744 |
| 2-oxoisocaproic acid | Keto acid | C6 H10 O3 | 130.063 | 816-66-0 | HMDB00695 |
| Adipic acid | Carboxylic acid | C6 H10 O4 | 146.058 | 124-04-9 | HMDB00448 |
| Citric acid | Carboxylic acid | C6 H8 O7 | 192.027 | 77-92-9 | HMDB00094 |
| Oleic acid | Fatty acid | C18 H34 O2 | 282.255 | 112-79-8 | HMDB00207 |
| Linolenic acid | Fatty acid | C18 H30 O2 | 278.224 | 463-40-1 | HMDB01388 |
| Gondoic acid | Fatty acid | C20 H38 O2 | 310.286 | 5561-99-9 | HMDB02231 |
| Docosahexaenoic acid | Fatty acid | C22 H32 O2 | 328.24 | 6217-54-5 | HMDB02183 |
| Docosapentaenoic acid | Fatty acid | C22 H34 O2 | 330.256 | 24880-45-3 | HMDB06528 |
| Myristic acid | Fatty acid | C14 H28 O2 | 228.208 | 544-63-8 | HMDB00806 |
| Palmitic acid | Fatty acid | C16 H32 O2 | 256.24 | 57-10-3 | HMDB00220 |
| Palmitoleic acid | Fatty acid | C16 H30 O2 | 254.225 | 373-49-9 | HMDB03229 |
| Stearic acid | Fatty acid | C18 H36 O2 | 284.27 | 57-11-4 | HMDB00827 |
| Hippuric acid | Benzamide | C7 H6 O3 | 138.032 | 495-69-2 | HMDB00714 |
| 4-Pyridoxic acid | Pyridine | C9 H9 N O3 | 179.058 | 82-82-6 | HMDB00017 |
| Tyrosine | Amino acid | C8 H9 N O4 | 183.053 | 556-03-6 | HMDB00158 |
| N-Acetyl-DL-methionine | Amino acid | C11 H12 N2 O2 | 204.09 | 1115-47-5 | HMDB11745 |
| N-Acetyl-L-alanine | Amino acid | C9 H11 N O3 | 181.074 | 97-69-8 | HMDB00766 |
| Pyroglutamic acid | Carboxylic acid | C9 H11 N O2 | 165.079 | 98-79-3 | HMDB00267 |
| Uric acid | Alkaloid | C7 H13 N O3 S | 191.061 | 69-93-2 | HMDB00289 |
| Chenodeoxycholic Acid | Cholic acid | C24 H40 O4 | 392.291 | 474-25-9 | HMDB00518 |
| Cholic acid | Cholic acid | C24 H40 O5 | 408.287 | 81-25-4 | HMDB00619 |
| Deoxycholic acid | Cholic acid | C24 H40 O4 | 392.292 | 83-44-3 | HMDB00626 |
| Chenodeoxycholic acid glycine conjugate | Cholic acid | C26 H43 N O5 | 449.314 | 640-79-9 | HMDB00637 |
| Glycocholic Acid | Cholic acid | C26 H43 N O6 | 465.308 | 475-31-0 | HMDB00138 |
| Glycodeoxycholate | Cholic acid | C26 H43 N O5 | 449.314 | 360-65-6 | HMDB00631 |
| Taurochenodeoxycholic acid | Cholic acid | C26 H45 N O6 S | 499.296 | 516-35-8 | HMDB00951 |
| Cortisone | Steroid | C21 H28 O5 | 360.193 | 53-06-5 | HMDB02802 |
| Uridine | Nucleoside | C7 H8 N4 O2 | 180.065 | 58-96-8 | HMDB00296 |
| Xanthine | Alkaloid | C9 H12 N2 O6 | 244.07 | 69-89-6 | HMDB00292 |
| LPC(18:0) | LysoPC | C26 H54 N O7 P | 523.364 | 19420-57-6 | HMDB10384 |
| Indoxylsulfuric acid | Indole | C8 H7 N O4 S | 213.01 | 61-19-8 | HMDB00682 |
| Threonate | Amino acid | C4 H8 O5 | 136.038 | 70753-61-6 | HMDB00943 |
| 2-Methylglutaric acid | Carboxylic acid | C6 H10 O4 | 146.058 | 617-62-9 | HMDB00422 |
| Beta-hydroxypalmitic acid | Fatty acid | C16 H32 O3 | 272.235 | 928-17-6 | HMDB10734 |
| Beta-hydroxylauric acid | Fatty acid | C12 H24 O3 | 216.175 | 53941-38-1 | HMDB00387 |
| Dodecanedioic acid | Fatty acid | C12 H22 O4 | 230.151 | 693-23-2 | HMDB00623 |
| Azelaic acid | Dicarboxylic acid | C9 H16 O4 | 188.105 | 123-99-9 | HMDB00784 |
| 3-Indolelactic acid | Indole | C11 H11 N O3 | 205.074 | 832-97-3 | HMDB00759 |

Table B. Lipid species detected in plasma biobank samples investigated in the study.

| **Compound name** | **Lipid class** | **Formula** | **Mass** |
| --- | --- | --- | --- |
| LPC(14:0) | Lysophosphatidylcholine | C22 H46 N O7 P | 467.303 |
| LPC(O-16:0) | Lysophosphatidylcholine | C24 H52 N O6 P | 481.354 |
| LPC(16:1) | Lysophosphatidylcholine | C24 H48 N O7 P | 493.318 |
| LPC(16:0) | Lysophosphatidylcholine | C24 H50 N O7 P | 495.334 |
| LPC(17:0) | Lysophosphatidylcholine | C25 H52 N O7 P | 509.349 |
| LPC(18:3) | Lysophosphatidylcholine | C26 H48 N O7 P | 517.318 |
| LPC(18:2) | Lysophosphatidylcholine | C26 H50 N O7 P | 519.333 |
| LPC(18:1) | Lysophosphatidylcholine | C26 H52 N O7 P | 521.349 |
| LPC(18:0) | Lysophosphatidylcholine | C26 H54 N O7 P | 523.365 |
| LPC(20:4) | Lysophosphatidylcholine | C28 H50 N O7 P | 543.331 |
| LPC(20:3) | Lysophosphatidylcholine | C28 H52 N O7 P | 545.349 |
| LPC(20:0) | Lysophosphatidylcholine | C28 H58 N O7 P | 551.396 |
| LPC(22:6) | Lysophosphatidylcholine | C30 H50 N O7 P | 567.333 |
| PC(30:1) | Phosphatidylcholine | C38 H74 N O8 P | 703.518 |
| PC(32:2) | Phosphatidylcholine | C40 H76 N O8 P | 729.532 |
| PC(32:1) | Phosphatidylcholine | C40 H78 N O8 P | 731.548 |
| PC(32:0) | Phosphatidylcholine | C40 H80 N O8 P | 733.562 |
| PC(O-34:3) | Phosphatidylcholine | C42 H80 N O7 P | 741.574 |
| PC(33:0) | Phosphatidylcholine | C41 H82 N O8 P | 747.577 |
| PC(34:4) | Phosphatidylcholine | C42 H76 N O8 P | 753.531 |
| PC(34:3) | Phosphatidylcholine | C42 H78 N O8 P | 755.548 |
| PC(34:2) | Phosphatidylcholine | C42 H80 N O8 P | 757.564 |
| PC(34:1) | Phosphatidylcholine | C42 H82 N O8 P | 759.58 |
| PC(O-36:5) | Phosphatidylcholine | C44 H80 N O7 P | 765.568 |
| PC(O-36:4) | Phosphatidylcholine | C44 H82 N O7 P | 767.583 |
| PC(35:2) | Phosphatidylcholine | C43 H82 N O8 P | 771.584 |
| PC(35:1) | Phosphatidylcholine | C43 H84 N O8 P | 773.595 |
| PC(36:6) | Phosphatidylcholine | C44 H76 N O8 P | 777.531 |
| PC(36:5) | Phosphatidylcholine | C44 H78 N O8 P | 779.548 |
| PC(36:4) | Phosphatidylcholine | C44 H80 N O8 P | 781.564 |
| PC(36:3) | Phosphatidylcholine | C44 H82 N O8 P | 783.579 |
| PC(36:2) | Phosphatidylcholine | C44 H84 N O8 P | 785.595 |
| PC(36:1) | Phosphatidylcholine | C44 H86 N O8 P | 787.611 |
| PC(O-38:5) | Phosphatidylcholine | C46 H84 N O7 P | 793.597 |
| PC(O-38:4) | Phosphatidylcholine | C46 H86 N O7 P | 795.615 |
| PC(38:6) | Phosphatidylcholine | C46 H80 N O8 P | 805.564 |
| PC(38:5) | Phosphatidylcholine | C46 H82 N O8 P | 807.579 |
| PC(38:4) | Phosphatidylcholine | C46 H84 N O8 P | 809.595 |
| PC(38:3) | Phosphatidylcholine | C46 H86 N O8 P | 811.611 |
| PC(40:8) | Phosphatidylcholine | C48 H80 N O8 P | 829.561 |
| PC(40:7) | Phosphatidylcholine | C48 H82 N O8 P | 831.578 |
| PC(40:6) | Phosphatidylcholine | C48 H84 N O8 P | 833.595 |
| PC(40:5) | Phosphatidylcholine | C48 H86 N O8 P | 835.61 |
| PC(40:4) | Phosphatidylcholine | C48 H88 N O8 P | 837.624 |
| SM(tot32:2) | Sphingomyelin | C37 H73 N2 O6 P | 672.521 |
| SM(tot32:1) | Sphingomyelin | C37 H75 N2 O6 P | 674.538 |
| SM(tot33:1) | Sphingomyelin | C38 H77 N2 O6 P | 688.553 |
| SM(tot34:2) | Sphingomyelin | C39 H77 N2 O6 P | 700.553 |
| SM(tot34:1) | Sphingomyelin | C39 H79 N2 O6 P | 702.569 |
| SM(tot34:0) | Sphingomyelin | C39 H81 N2 O6 P | 704.583 |
| SM(tot36:2) | Sphingomyelin | C41 H81 N2 O6 P | 728.586 |
| SM(tot36:1) | Sphingomyelin | C41 H83 N2 O6 P | 730.603 |
| SM(tot38:2) | Sphingomyelin | C43 H85 N2 O6 P | 756.612 |
| SM(tot38:1) | Sphingomyelin | C43 H87 N2 O6 P | 758.631 |
| SM(tot39:1) | Sphingomyelin | C44 H89 N2 O6 P | 772.645 |
| SM(tot40:2) | Sphingomyelin | C45 H89 N2 O6 P | 784.647 |
| SM(tot41:2) | Sphingomyelin | C46 H91 N2 O6 P | 798.662 |
| SM(tot42:3) | Sphingomyelin | C47 H91 N2 O6 P | 810.662 |
| DG(34:1) | Diacyglycerol | C37 H70 O5 | 594.523 |
| DG(36:4) | Diacyglycerol | C39 H68 O5 | 616.505 |
| DG(36:2) | Diacyglycerol | C39 H72 O5 | 620.538 |
| DG(38:5) | Diacyglycerol | C41 H70 O5 | 642.52 |
| PA(34:0) | Phosphatidic acid | C37 H73 O8 P | 676.505 |
| PA(36:3) | Phosphatidic acid | C39 H71 O8 P | 698.488 |
| PA(38:6) | Phosphatidic acid | C41 H69 O8 P | 720.47 |
| PE(34:0) | Phosphatidylethanolamine | C39 H78 N O8 P | 719.546 |
| PE(36:5) | Phosphatidylethanolamine | C41 H74 N O7 P | 723.527 |
| PE(36:2) | Phosphatidylethanolamine | C41 H76 N O8 P | 741.53 |
| PE(38:5) | Phosphatidylethanolamine | C43 H78 N O7 P | 751.556 |
| PE(38:4) | Phosphatidylethanolamine | C43 H74 N O8 P | 763.515 |
| PG(40:4) | Phosphatidylglycerol | C46 H83 O10 P | 826.572 |
| PS(38:5) | Phosphatidylserine | C44 H76 N O10 P | 809.514 |
| PI(32:1) | Phosphatidylinositol | C41 H77 O13 P | 808.51 |
| TG(40:0) | Triglyceride | C43 H82 O6 | 694.615 |
| TG(42:2) | Triglyceride | C45 H82 O6 | 718.612 |
| TG(42:1) | Triglyceride | C45 H84 O6 | 720.63 |
| TG(42:0) | Triglyceride | C45 H86 O6 | 722.648 |
| TG(44:2) | Triglyceride | C47 H86 O6 | 746.644 |
| TG(44:1) | Triglyceride | C47 H88 O6 | 748.66 |
| TG(44:0) | Triglyceride | C47 H90 O6 | 750.677 |
| TG(45:1) | Triglyceride | C48 H90 O6 | 762.677 |
| TG(46:2) | Triglyceride | C49 H90 O6 | 774.675 |
| TG(46:1) | Triglyceride | C49 H92 O6 | 776.691 |
| TG(46:0) | Triglyceride | C49 H94 O6 | 778.706 |
| TG(47:2) | Triglyceride | C50 H92 O6 | 788.687 |
| TG(47:0) | Triglyceride | C50 H96 O6 | 792.722 |
| TG(48:4) | Triglyceride | C51 H90 O6 | 798.674 |
| TG(48:3) | Triglyceride | C51 H92 O6 | 800.691 |
| TG(48:2) | Triglyceride | C51 H94 O6 | 802.707 |
| TG(48:1) | Triglyceride | C51 H96 O6 | 804.723 |
| TG(48:0) | Triglyceride | C51 H98 O6 | 806.737 |
| TG(49:2) | Triglyceride | C52 H96 O6 | 816.722 |
| TG(49:1) | Triglyceride | C52 H98 O6 | 818.737 |
| TG(49:0) | Triglyceride | C52 H100 O6 | 820.753 |
| TG(50:4) | Triglyceride | C53 H94 O6 | 826.706 |
| TG(50:3) | Triglyceride | C53 H96 O6 | 828.723 |
| TG(50:2) | Triglyceride | C53 H98 O6 | 830.739 |
| TG(50:1) | Triglyceride | C53 H100 O6 | 832.753 |
| TG(50:0) | Triglyceride | C53 H102 O6 | 834.77 |
| TG(51:4) | Triglyceride | C54 H96 O6 | 840.723 |
| TG(51:3) | Triglyceride | C54 H98 O6 | 842.738 |
| TG(51:2) | Triglyceride | C54 H100 O6 | 844.754 |
| TG(51:1) | Triglyceride | C54 H102 O6 | 846.77 |
| TG(52:5) | Triglyceride | C55 H96 O6 | 852.722 |
| TG(52:4) | Triglyceride | C55 H98 O6 | 854.739 |
| TG(52:3) | Triglyceride | C55 H100 O6 | 856.755 |
| TG(52:2) | Triglyceride | C55 H102 O6 | 858.771 |
| TG(52:1) | Triglyceride | C55 H104 O6 | 860.787 |
| TG(52:0) | Triglyceride | C55 H106 O6 | 862.799 |
| TG(53:5) | Triglyceride | C56 H98 O6 | 866.74 |
| TG(53:4) | Triglyceride | C56 H100 O6 | 868.755 |
| TG(53:3) | Triglyceride | C56 H102 O6 | 870.772 |
| TG(54:9) | Triglyceride | C57 H92 O6 | 872.688 |
| TG(53:2) | Triglyceride | C56 H104 O6 | 872.786 |
| TG(53:1) | Triglyceride | C56 H106 O6 | 874.801 |
| TG(54:7) | Triglyceride | C57 H96 O6 | 876.721 |
| TG(54:5) | Triglyceride | C57 H100 O6 | 880.756 |
| TG(54:4) | Triglyceride | C57 H102 O6 | 882.773 |
| TG(54:3) | Triglyceride | C57 H104 O6 | 884.788 |
| TG(54:2) | Triglyceride | C57 H106 O6 | 886.803 |
| TG(54:1) | Triglyceride | C57 H108 O6 | 888.816 |
| TG(54:0) | Triglyceride | C57 H110 O6 | 890.829 |
| TG(55:3) | Triglyceride | C58 H106 O6 | 898.805 |
| TG(56:9) | Triglyceride | C59 H96 O6 | 900.72 |
| TG(56:8) | Triglyceride | C59 H98 O6 | 902.738 |
| TG(56:7) | Triglyceride | C59 H100 O6 | 904.754 |
| TG(56:6) | Triglyceride | C59 H102 O6 | 906.769 |
| TG(56:2) | Triglyceride | C59 H110 O6 | 914.831 |
| TG(56:1) | Triglyceride | C59 H112 O6 | 916.845 |
| TG(58:11) | Triglyceride | C61 H96 O6 | 924.723 |
| TG(58:10) | Triglyceride | C61 H98 O6 | 926.737 |
| TG(58:9) | Triglyceride | C61 H100 O6 | 928.752 |
| TG(58:8) | Triglyceride | C61 H102 O6 | 930.768 |
| TG(58:7) | Triglyceride | C61 H104 O6 | 932.784 |
| TG(58:6) | Triglyceride | C61 H106 O6 | 934.808 |
| TG(58:2) | Triglyceride | C61 H114 O6 | 942.861 |
| TG(60:13) | Triglyceride | C63 H96 O6 | 948.719 |
| TG(60:12) | Triglyceride | C63 H98 O6 | 950.737 |
| TG(60:3) | Triglyceride | C63 H116 O6 | 968.876 |
| TG(60:2) | Triglyceride | C63 H118 O6 | 970.892 |
